# Clinical Outcomes Following Dose-Escalated Proton Therapy for Skull-Base Chordoma

**DOI:** 10.14338/IJPT-20-00066.1

**Published:** 2021-06-25

**Authors:** Adam L. Holtzman, Ronny L. Rotondo, Michael S. Rutenberg, Daniel J. Indelicato, Alexandra De Leo, Dinesh Rao, Jeet Patel, Christopher G. Morris, William M. Mendenhall

**Affiliations:** 1 Department of Radiation Oncology University of Florida College of Medicine, Jacksonville, FL, USA; 2 Department of Radiology, University of Florida College of Medicine, Jacksonville, FL, USA; 3 Department of Radiation Oncology, University of Kansas, Kansas City, KS, USA

**Keywords:** radiation oncology, proton therapy, particle therapy, skull-based tumors, chordoma, head and neck

## Abstract

**Purpose:**

To evaluate the effectiveness of external-beam proton therapy (PT) on local control and survival in patients with skull-base chordoma.

**Materials and Methods:**

We reviewed the medical records of patients with skull-base chordoma treated with definitive or adjuvant high-dose PT and updated their follow-up when feasible. We assessed overall survival, disease-specific survival, local control, and freedom from distant metastasis. Radiotherapy toxicities were scored using the Common Terminology Criteria for Adverse Events, version 4.0.

**Results:**

A total 112 patients were analyzed, of whom 105 (94%) received PT and 7 (6%) received combined proton-photon therapy between 2007 and 2019. Eighty-seven patients (78%) underwent a subtotal resection, 22 (20%) a gross total resection, and 3 (3%) a biopsy alone. The median radiotherapy dose was 73.8 Gy radiobiologic equivalent (GyRBE; range, 69.6-74.4). Ninety patients (80%) had gross disease at radiotherapy and 7 (6%) were treated for locally recurrent disease following surgery. Median follow-up was 4.4 years (range, 0.4-12.6); for living patients, it was 4.6 years (range, 0.4-12.6), and for deceased patients, 4.1 years (range, 1.2-11.2). At 5 years after radiotherapy, the actuarial overall survival, disease-specific survival, local control, and freedom from distant metastasis rates were 78% (n = 87), 83% (n = 93), 74% (n = 83), and 99% (n = 111), respectively. The median time to local progression was 2.4 years (range, 0.8-7). Local control and disease-specific survival by resection status was 95% versus 70% (*P* = 0.28) and 100% versus 80% (*P* = 0.06) for gross total, versus subtotal, resection or biopsy alone, respectively. There were no serious acute toxicities (grade ≥ 3) related to radiotherapy.

**Conclusion:**

High-dose PT alone or after surgical resection for skull-base chordoma reaffirms the favorable 5-year actuarial local control rate compared with conventional techniques with acceptable late-complication–free survival. Outcomes following gross total resection and adjuvant PT were excellent. Further follow-up of this cohort is necessary to better characterize long-term disease control and late toxicities.

## INTRODUCTION

Chordomas are a rare group of neoplasms that arise from the remnant embryologic notochord. They can occur anywhere along the vertebral axis, spanning cranially from the upper clivus and caudally to the sacrococcygus. Optimal management is maximal safe surgery with the intent of gross total resection or optimization of target geometry for postoperative high-dose radiotherapy. In contrast to skull-base chondrosarcomas, which tend to arise from paramedian structures and have a favorable long-term prognosis, chordomas, although typically indolent, are radioresistant tumors of the median neurovertebral axis that have a significantly lower 5- and 10-year local control (LC) [1].

Because these tumors require radiation doses > 70 Gy radiobiologic equivalent (GyRBE) for disease control, conventional radiotherapy techniques in the past were limited because of their proximity to dose-limiting neural structures, such as the brainstem, spinal cord, and optic apparatus. Modern radiotherapy (RT) techniques, such as static beam angle and rotational intensity-modulated RT (IMRT), stereotactic radiosurgery, proton therapy (PT), and carbon therapy have allowed for RT-dose escalation, which has led to better treatment outcomes [2]. Herein, we report outcomes of patients treated at a single institution with dose-escalated PT for skull-base chordoma.

## METHODS

Under institutional review board approval, with patient informed consent, we reviewed the medical records of patients enrolled on a prospective outcomes-tracking protocol who were treated with high-dose PT for skull-base chordomas at our institution and who met the following inclusion criteria: primary site with its epicenter arising from the base of the skull (sphenoid, clivus, petrous, or basiocciput), no history of prior irradiation or evidence of metastatic disease, age 22 years or older at the time of consent, Karnofsky performance status ≥ 50, and surgery ranging from biopsy to gross total resection before PT. The age cutoff was chosen in keeping with many cooperative group trials that include patients up to age 22 and serves to demarcate the adult and pediatric teams for clinical and research purposes at our institution. *Recurrent disease*, for the purposes of this study, was defined as disease progression after initial surgical-alone management.

Because our institute is a regional and international referral center, most follow-up assessments were conducted remotely, aided by the referring home team. We recommended that participants be followed every 3 months for the first 2 years, every 6 months up to 5 years, then annually, thereafter, with basic examination and imaging and annual audiology, ophthalmology, and endocrine testing when indicated based on patient-specific doses to the organs at risk (OARs). Neuroimaging was reviewed at a multidisciplinary conference for treatment planning and, when indicated, with radiographic follow-up [3].

Statistical analysis was performed with JMP Pro 13.0 (SAS Institute, Cary, North Carolina). The Kaplan-Meier product limit method provided estimates of overall survival (OS), disease-specific survival (DSS), LC, and freedom from distant metastasis (FFDM). The log-rank test statistic assessed differences between gross and subtotal resections for those endpoints. The PT-related toxicities were scored using the National Cancer Institute's Common Terminology Criteria for Adverse Events, version 4.0.

Techniques for computed tomography simulation, planning, and dosimetric evaluation for target coverage, heterogeneity goals, and dose constraints have been previously described [1, 4, 5]. Consistent with institutional approach, composite dose under coverage of target volumes was permitted if necessary to meet brainstem, optic chiasm, bilateral optic nerve, and spinal cord absolute constraints. Unilateral optic nerve, temporal lobe, retina, and bilateral cochlear constraints were exceeded on a case-by-case basis.

## RESULTS

**Figure 1.**
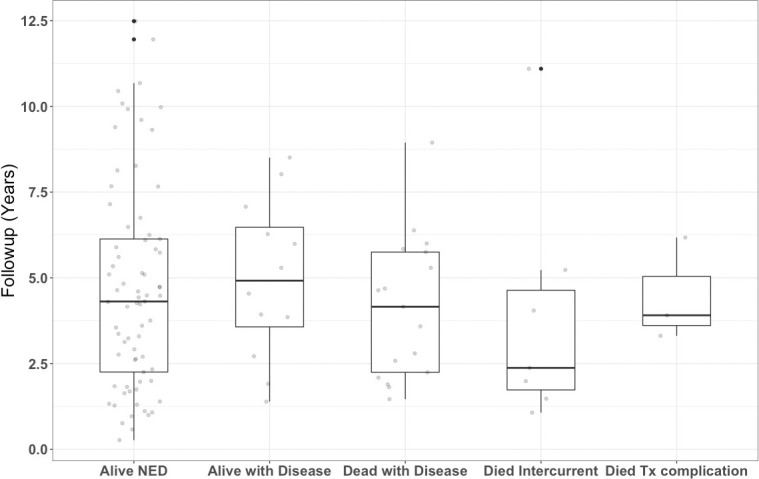
Boxplot overlaid with individual follow-up timepoints by disease status. Abbreviations: NED, no evidence of disease; TX, treatment.

In total, 112 patients treated between February 2007 and October 2019 were eligible for analysis. Patient characteristics and presenting symptoms are detailed in Table 1. Nearly all patient follow-up was updated within a minimum of 1 year of analysis and the duration of follow-up is detailed in Figure 1 with both boxplots and plotted individual-specific time references. The median follow-up was 4.4 years (range, 0.4-12.6); it was 4.6 years (range, 0.4-12.6) for living patients and 4.1 years (range, 1.2-11.2) for deceased patients. All patients in the present series had conventional histologic classification. Treatment and tumor details are shown in Table 2**.**

**Table 1.** Patient Characteristics (N = 112)

**Characteristic**	**No. (%)**
Age, y, median (range)	52 (22-78)
Sex	
Women	35 (31)
Men	77 (69)
Race	
Asian/Pacific	6 (5)
Black	6 (5)
Hispanic	7 (6)
White	93 (83)
Comorbid conditions	
Hypertension	51 (45)
Smoking history (> 10 pack-y)	38 (34)
Hyperlipidemia	29 (26)
Diabetes	10 (9)
Cardiac disease	9 (8)
Presenting symptoms	
Diplopia	55 (49)
Headaches	48 (43)
Dysphagia/dysarthria	16 (14)
Incidental	9 (8)
Facial weakness/numbness/pain	9 (8)
Neck pain	9 (8)
Imbalance/vertigo	8 (7)
Sinusitis/nasal congestion	6 (5)
Visual acuity changes	4 (4)
Loss of consciousness	3 (3)
Hearing loss	3 (3)

**Table 2.** Tumor and Treatment Characteristics (N = 112)

**Characteristic**	**No. (%)**
Recurrent^a^	
No	105 (95)
Yes	7 (5)
Resection status	
Subtotal	87 (78)
Gross total	22 (20)
Biopsy	3 (3)
Involved site	
Clivus	112 (100)
Sphenoid bone	94 (84)
Cavernous sinus	81 (72)
Suprasellar	61 (54)
Petrous bone	60 (54)
Ethmoid	20 (18)
Cervical spine	10 (9)
Modality	
Proton	104 (93)
Proton + photon	8 (7)
Dose, GyRBE, median (range)	73.8 (69.6-75.6)
Fractionation	
Once daily	99 (88)
Twice daily	13 (12)

a *Recurrent* was defined as initially managed with surgery alone and radiotherapy delivered after documented disease progression.

**Abbreviations:** GyRBE, Gy radiobiologic equivalent; RT, radiotherapy.

Ninety-seven patients (87%) were treated with a double-scattered PT and 6 (5%) with the pencil-beam technique alone. One patient (0.9%) received combined double-scattered and pencil-beam treatment. Eight patients (7%) received a component of IMRT; of which, all but 1 was combined with double-scattered proton therapy. The median photon dose received was 23.4 Gy (range, 1.8-30.6 Gy), with 3 patients receiving < 7.2 Gy via IMRT because of maintenance of the proton cyclotron.

**Figure 2.**
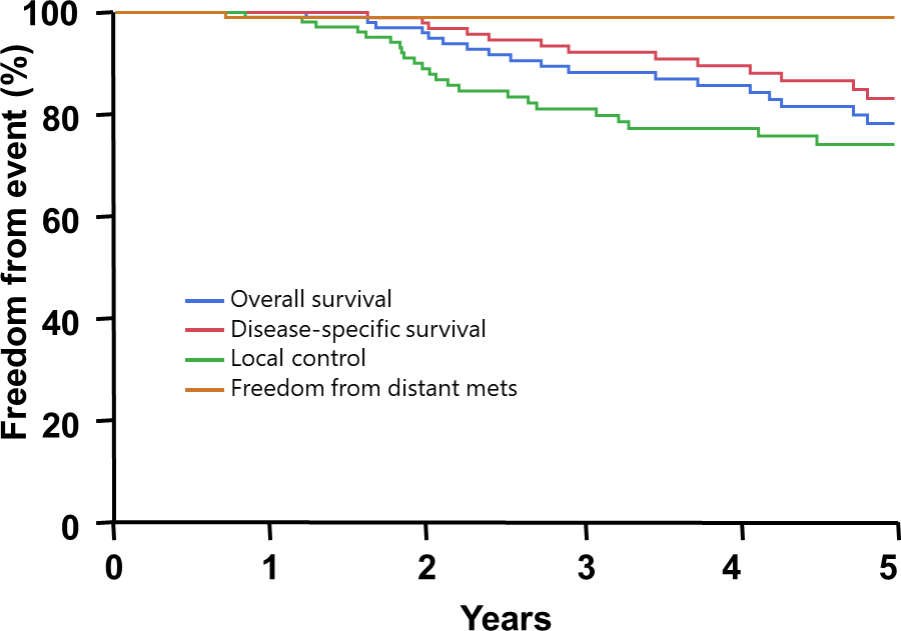
Oncologic endpoints at 5 years as measured by percentage and in years.

**Figure 3.**
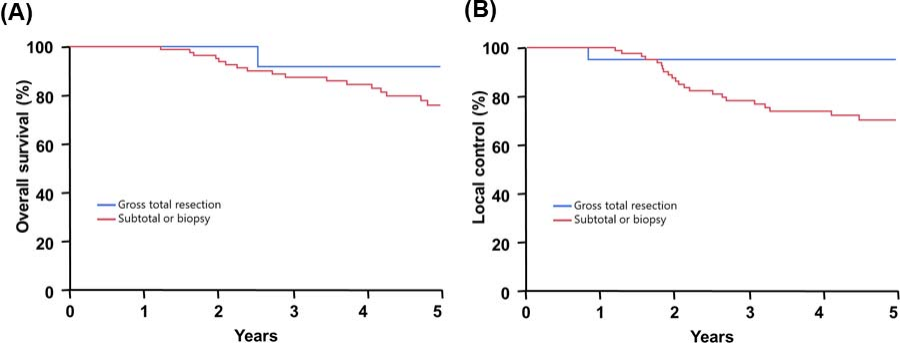
(A) Local-control and (B) cause-specific survival by resection status as measured by percentage and in years.

**Figure 4.**
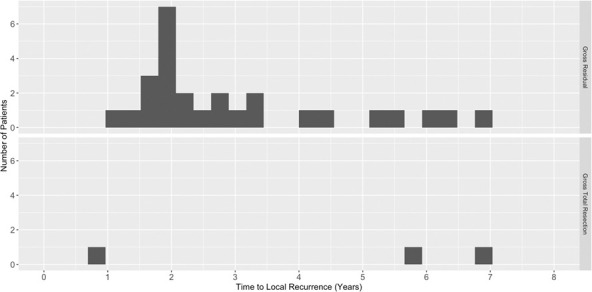
Time to local recurrence as defined by disease progression by resection status measured in years from radiation initiation.

The disease control and survival outcomes are shown in Figure 2. At 5 years after RT, the actuarial rates of OS, DSS, LC, and FFDM were 78%, 83%, 74%, and 99%, respectively. As defined by resection status, for those with a gross total resection, the 5-year rates of OS, DSS, and LC were 92%, 100%, and 95%, respectively. This was not statistically different as compared with OS, DSS, and LC rates among those who received a subtotal resection or biopsy, which were 76% (*P* = 0.28), 80% (*P* = 0.06), and 70% (*P* = 0.28), respectively. Comparisons by resection status are shown in Figure 3. The median time to local progression was 2.4 years (range, 0.8-7 years). Figure 4 illustrates the time to progression in years as subdivided by resection status.

The median time to recurrence by resection status was 2.2 years after subtotal resection and biopsy alone, and 5.8 years after gross total resection. Local salvage, which included further resection or radiosurgery, was not attempted in 8 patients (7%), attempted but not successful in 12 (11%), and attempted and successful to date in 10 (9%) patients. *Successful salvage* was defined as no current evidence of further disease progression since the salvage intervention. One patient (0.8%), who did not have total spine imaging pretreatment, was found to have a separate site of chordoma within the spine within 2 months of treatment.

The PT-related complications were categorized as *acute* or *late* events. All patients (100%; 112 of 112) had varying degrees and combinations of acute grade 1 and 2 toxicities during PT, which included fatigue, radiation dermatitis, alopecia, nausea, and mucositis. There were no (0%; 0 of 112) grade ≥ 3 acute toxicities attributed to PT, and late complications were classified by OARs. At least 17% (n = 19) of patients developed hypopituitarism requiring supplementation. One patient (0.8%) was hospitalized because of dehydration and was identified to have hypopituitarism including central adrenal insufficiency. Five percent (n = 6) of patients had worsening of baseline hearing with hearing aids indicated; < 3% (n = 3) of patients had grade ≥ 3 vision loss. Two patients (2%) had bilateral useful vision loss, and 1 patient (0.8%) had an ipsilateral complete vision loss. Five percent (n = 6) of patients had symptomatic grade 3 temporal injuries: 4 had ipsilateral and 2 had bilateral injury, for which multiple therapies, including steroids, bevacizumab, surgical resection, or long-term antiepileptics, were needed for management. An additional 4% (n = 5) of patients had asymptomatic or mildly symptomatic grade 2 temporal lobe necrosis for which steroids were indicated based on radiographic findings but were otherwise managed conservatively. One patient (0.8%) had a left internal carotid vascular event causing a cerebrovascular accident after treatment. Two patients (2%) had complications from attempted salvage surgery after a local recurrence, including 1 grade 4 cerebrovascular accident leading to feeding-tube dependence and 1 death (grade 5) after meningitis. Lastly, there were 2 (2%) grade 5 events potentially attributed to the radiotherapy: 1 patient had a cavernoma with an intracerebral hemorrhage and another had an anaphylactic antibiotic reaction for treatment of a skull-base abscess. There were no grade ≥ 2 brainstem injuries.

## DISCUSSION

Maximal safe surgery to relieve symptoms related to mass effect or to improve the target geometry of highly conformal RT is the preferred initial approach to skull-base conventional chordomas. Not only does that provide a histologic diagnosis but also can relieve compression of critical structures and optimize postoperative RT. Given the tendency of chordomas to originate in median bony sites, surgeons have increasingly approached this subset using anterior endoscopic procedures, at times combined with a craniotomy or expanded approach if lateral or posterior access is needed. These practices have resulted in improved extent of resection, and the shift in surgical management parallels technologic advancements in the field of radiation oncology, providing greater ability to deliver highly conformal adjuvant radiotherapy [6–8].

The benefits of a greater extent of resection are multifactorial in that it allows the radiation oncologist to safely escalate the dose in the adjuvant setting and minimize the probability of complications through improved target geometry. Although likely underpowered to show a difference within the present analysis, LC and DSS rates at 5 years were 95% (*P* = 0.28) and 100% (*P* = 0.06) for those with a gross resection, compared with 70% and 76%, respectively, for those with an incomplete resection. As shown in Figure 4, most recurrences occurred within the first 3 years of follow-up. The 1 patient (5%; 1 of 22) who failed before 5 years in the gross total resection cohort was treated after a surgical-alone recurrence as compared with others undergoing de novo adjuvant therapy. Although the small number of patients treated with recurrent disease within the present series prohibits more sophisticated analysis, investigators have noted that the highest rates of LC are for those treated with radiotherapy in the primary setting [9, 10].

For patients with residual disease and optimal displacement from critical healthy structures, dose intensification with modern RT techniques, such as PT, has been used in the treatment of skull-base chordomas to improve LC. Because the dose tolerances of the adjacent sensitive neurovascular OARs are less than the dose required to control gross disease, even with the most-advanced technology, a minimum separation of at least 1 to 2 mm between the residual disease and OARs is necessary for adequate target coverage, sometimes more for certain structures, such as the spinal cord and optic apparatus.

Early reports indicate that PT provides advantages compared with conventional photon RT in delivering an adequate radiation dose to the tumor and reducing the dose to healthy tissues. As described in prior literature reviews and investigations, in the modern proton experience of chordoma treatment, the 3- to 5-year LC rates range from approximately 70% to 85% [1, 11–15]. Our cohort is comparable to those contemporary proton series with a 5-year LC of 74% (n = 83), although patients undergoing a macroscopic resection had a 5-year LC rate of 95% with a 100% (22 of 22) DSS rate. Fung et al [15] reported a similar 5-year LC rate of 75% and suggested gross tumor volume > 25 mL was an unfavorable prognostic factor in LC. Investigators at the Paul Scherrer Institute (Villigen, Switzerland) reported a 7-year LC rate of 71% in 71 patients [14].

An additional factor to consider in our analysis is that nearly all surgeries were not performed at our medical center. This is an important reflection when comparing results across institutions in that favorable intermediate-term outcomes after PT when performed at a high-volume proton center can be achieved, even with wide heterogeneity in the surgical center [11, 13]. Having a radiation oncologist involved early in the surgical planning can assist with defining the areas of interest that achieve the greatest benefit from postsurgical target-geometry optimization [16]. However, given the relative scarcity of proton centers worldwide, it is inevitable that many patients will continue to require referral to an outside institution for PT. In that regard, these results are encouraging because our pragmatic model of close dialogue and coordination with outside surgical teams can offset concerns of compromised survival.

The present series reaffirms prior series that salvage was poor after recurrence. There are current practice guidelines and some early, emerging reported experiences with salvage therapy [17]. Raza et al [18] reviewed 29 patients treated at the MD Anderson Cancer Center (Houston, Texas). With postradiotherapy local failure, repeat resection did not confer any benefit (13.5 versus 17.6 months; *P* > .05) and, although not statistically significant, those who received stereotactic radiosurgery experienced prolonged survival (28.3 versus 16.2 months, *P* = .233). As noted by Beer et al [19] in a series published by the Paul Scherrer Institute, survival of patients with chordoma after a treatment failure following PT was poor. They found that 75% of patients ultimately died, with a median OS of 3.4 years after treatment failure. Within the present series, two thirds of patients (20 of 30; 67%%) with locally recurrent disease after treatment either did not attempt salvage (8 of 30; 27%) or were unsuccessful (12 of 30; 40%) without further disease progression to date. It is clear that more innovative options are still needed for this population [20, 21].

Regarding treatment complications, and corroborating other publications, such as that by Sahgal et al [22] and others [15, 23], there were no brainstem injuries, despite delivering doses upward of 64 GyRBE to the surface of the brainstem. Of note, this finding distinctly differs from the pediatric literature: Indelicato et al [24] showed that the cumulative incidence of serious brainstem toxicity was 2.1% at even lower dose thresholds in children with brain tumors. The difference likely reflects the sensitivity of the developing pediatric brainstem to ionizing radiation or disease-specific differences in surgical approach. Overall, the risk of symptomatic brainstem injury is reported as lower among adult patients treated with high-dose PT for skull-base tumors [25].

We observed several grade 3 temporal lobe radionecrosis events, with a crude incidence of 5% (6 of 112). This rate was slightly higher than the 2% reported by Fung et al [15], which included 1 grade 5 event. Our results resemble those of Weber et al [14], who observed a toxicity rate of 5%, with temporal lobe radionecrosis being the most common grade 3 complication reported. McDonald et al [26] reviewed the outcomes of 66 patients treated for skull-base malignancies and reported a 15% 3-year risk of any-grade temporal lobe radiation necrosis when the absolute volume of a temporal lobe receiving 60 GyRBE exceeded 5.5 cm^3^ or 70 GyRBE exceeded 1.7 cm^3^. Other groups have shown similar thresholds, such as using a dose of 62 GyRBE to 2 cm^3^ as the relative OAR tolerance dose for planning constraints [27, 28]. Although the present rate of temporal lobe injury is less than that found in IMRT series on other advanced skull-base tumors [29–31], further analysis is forthcoming from our group analyzing the relationship of temporal lobe dose to other potential contributing factors.

No patient in the present series received upfront induction or planned adjuvant chemotherapy as patients with poorly differentiated tumors were excluded from analysis. Chemotherapy or targeted systemic therapy was reserved for those with recurrent disease. Although multimodality therapy incorporating upfront chemotherapy, radiotherapy, and surgery for poorly differentiated tumors may improve oncologic disease control, that issue was not assessed by the present series [32]. Data analyzing recurrent and metastatic disease are beginning to emerge, and, given the poor prognosis with tumor recurrence, future research should help determine which agents are the most efficacious for the development of novel agents [20, 21].

Our study is not without its limitations. Although we analyzed a longitudinal cohort of patients prospectively enrolled in an outcomes-tracking protocol, the present analysis was retrospectively performed. Direct contact is maintained with both patients and referring physicians through toxicity and outcomes assessments via an electronic patient portal, relevant imaging was reviewed by our team, and a high level of continued follow-up was maintained, as shown in Figure 1. We are, therefore, confident in our estimates of disease control and clinically meaningful late complications. However, as most follow-up was performed remotely, we recognize the potential for inconsistent reporting of grade 1 and 2 toxicity. Additionally, a median follow-up under 5 years is insufficient time to fully characterize the recurrence and outcome patterns. These issues, including the heterogeneity of the study population, are intrinsic to most rare disease entities treated at referral centers, wherein travel for continued follow-up at the treating institution is challenging for logistical or financial reasons. However, this shortcoming is not isolated to the present series. Investigators at our institution have found that disease control is not compromised when multidisciplinary teams are willing to coordinate complex patient care across physical distance [11, 13, 15]. Although the present LC and DSS rate are promising, continued follow-up will be needed to determine long-term outcomes.

In summary, the present series reaffirms the contemporary proton experience that high-dose PT alone or after maximal safe surgical resection for skull-base chordoma provides improved LC compared with historic series, with acceptable late-complication–free survival, and no serious acute (grade ≥ 3) complications. The excellent LC and DSS rates observed among our patients underscore the value of safe macroscopic tumor resection. Further follow-up of this cohort is necessary to better characterize long-term disease control and late toxicities.

## References

[1] Mercado CE, Holtzman AL, Rotondo R, Rutenberg MS, Mendenhall WM (2019). Proton therapy for skull base tumors: a review of clinical outcomes for chordomas and chondrosarcomas. *Head Neck*.

[2] Palm RF, Oliver DE, Yang GQ, Abuodeh Y, Naghavi AO, Johnstone PAS (2019). The role of dose escalation and proton therapy in perioperative or definitive treatment of chondrosarcoma and chordoma: an analysis of the National Cancer Data Base. *Cancer*.

[3] Rao D, Fiester P, Patel J, Rutenberg M, Holtzman A, Dagan R, Rotondo RL, Sandhu SJS (2019). Multidisciplinary imaging review conference improves neuro-oncology radiation treatment planning and follow-up. *Cureus*.

[4] Holtzman AL, Rotondo RL, Rutenberg MS, Indelicato DJ, Mercado CE, Rao D, Tavanaiepour D, Morris CG, Louis D, Flampouri S, Mendenhall WM (2019). Proton therapy for skull-base chondrosarcoma, a single-institution outcomes study. *J Neurooncol*.

[5] Deraniyagala RL, Yeung D, Mendenhall WM, Li Z, Morris CG, Mendenhall NP, Okunieff P, Malyapa RS (2014). Proton therapy for skull base chordomas: an outcome study from the university of Florida proton therapy institute. *J Neurol Surg B Skull Base*.

[6] Zoli M, Milanese L, Bonfatti R, Faustini-Fustini M, Marucci G, Tallini G, Zenesini C, Sturiale C, Frank G, Pasquini E, Mazzatenta D (2018). Clival chordomas: considerations after 16 years of endoscopic endonasal surgery. *J Neurosurg*.

[7] Younus I, Gerges MM, Uribe-Cardenas R, Morgenstern PF, Eljalby M, Tabaee A, Greenfield JP, Kacker A, Anand VK, Schwartz TH (2020]). How long is the tail end of the learning curve? Results from 1000 consecutive endoscopic endonasal skull base cases following the initial 200 cases [published online ahead of print February 7. *J Neurosurg*.

[8] Snyderman CH, Gardner PA (2020). Current opinion in otolaryngology and head and neck surgery: clival chordoma and its management. *Curr Opin Otolaryngol Head Neck Surg*.

[9] Hug EB, Fitzek MM, Liebsch NJ, Munzenrider JE (1995). Locally challenging osteo- and chondrogenic tumors of the axial skeleton: results of combined proton and photon radiation therapy using three-dimensional treatment planning. *Int J Radiat Oncol Biol Phys*.

[10] Pennicooke B, Laufer I, Sahgal A, Varga PP, Gokaslan ZL, Bilsky MH, Yamada YJ (2016). Safety and local control of radiation therapy for chordoma of the spine and sacrum: a systematic review. *Spine (Phila Pa 1976)*.

[11] Munzenrider JE, Liebsch NJ (1999). Proton therapy for tumors of the skull base. *Strahlenther Onkol*.

[12] Hug EB, Loredo LN, Slater JD, DeVries A, Grove RI, Schaefer RA, Rosenberg AE, Slater JM (1999). Proton radiation therapy for chordomas and chondrosarcomas of the skull base. *J Neurosurg*.

[13] Grosshans DR, Zhu XR, Melancon A, Allen PK, Poenisch F, Palmer M, McAleer MF, McGovern SL, Gillin M, DeMonte F, Chang EL, Brown PD, Mahajan A (2014). Spot scanning proton therapy for malignancies of the base of skull: treatment planning, acute toxicities, and preliminary clinical outcomes. *Int J Radiat Oncol Biol Phys*.

[14] Weber DC, Malyapa R, Albertini F, Bolsi A, Kliebsch U, Walser M, Pica A, Combescure C, Lomax AJ, Schneider R (2016). Long term outcomes of patients with skull-base low-grade chondrosarcoma and chordoma patients treated with pencil beam scanning proton therapy. *Radiother Oncol*.

[15] Fung V, Calugaru V, Bolle S, Mammar H, Alapetite C, Maingon P, De Marzi L, Froelich S, Habrand JL, Dendale R, Noel G, Feuvret L (2018). Proton beam therapy for skull base chordomas in 106 patients: a dose adaptive radiation protocol. *Radiother Oncol*.

[16] Connors SW, Aoun SG, Shi C, Peinado-Reyes V, Hall K, Bagley CA (2020). Recent advances in understanding and managing chordomas: an update. *F1000Res*.

[17] Stacchiotti S, Gronchi A, Fossati P, Akiyama T, Alapetite C, Baumann M, Blay JY, Bolle S, Boriani S, Bruzzi P, Capanna R, Caraceni A, Casadei R, Colia V, Debus J, Delaney T, Desai A, Dileo P, Dijkstra S, Doglietto F, Flanagan A, Froelich S, Gardner PA, Gelderblom H, Gokaslan ZL, Haas R, Heery C, Hindi N, Hohenberger P, Hornicek F, Imai R, Jeys L, Jones RL, Kasper B, Kawai A, Krengli M, Leithner A, Logowska I, Martin Broto J, Mazzatenta D, Morosi C, Nicolai P, Norum OJ, Patel S, Penel N, Picci P, Pilotti S, Radaelli S, Ricchini F, Rutkowski P, Scheipl S,, Sen C, Tamborini E, Thornton KA, Timmermann B, Torri V, Tunn PU, Uhl M, Yamada Y, Weber DC, Vanel D, Varga PP, Vleggeert-Lankamp CLA, Casali PG, Sommer J (2017). Best practices for the management of local-regional recurrent chordoma: a position paper by the Chordoma Global Consensus Group. *Ann Oncol*.

[18] Raza SM, Bell D, Freeman JL, Grosshans DR, Fuller GN, DeMonte F (2018). Multimodality management of recurrent skull base chordomas: factors impacting tumor control and disease-specific survival. *Oper Neurosurg (Hagerstown)*.

[19] Beer J, Kountouri M, Kole AJ, Murray FR, Leiser D, Kliebsch U, Combescure C, Pica A, Bachtiary B, Bolsi A, Lomax AJ, Walser M, Weber DC (2020). Outcomes, prognostic factors and salvage treatment for recurrent chordoma after pencil beam scanning proton therapy at the Paul Scherrer Institute. *Clin Oncol (R Coll Radiol)*.

[20] Edem I, DeMonte F, Raza SM (2020]). Advances in the management of primary bone sarcomas of the skull base [published online ahead of print April 18. *J Neurooncol*.

[21] Hoffman SE, Al Abdulmohsen SA, Gupta S, Hauser BM, Meredith DM, Dunn IF, Bi WL (2020). Translational windows in chordoma: a target appraisal. *Front Neurol*.

[22] Sahgal A, Chan MW, Atenafu EG, Masson-Cote L, Bahl G, Yu E, Millar BA, Chung C, Catton C, O'Sullivan B, Irish JC, Gilbert R, Zadeh G, Cusimano M, Gentili F, Laperriere NJ (2015). Image-guided, intensity-modulated radiation therapy (IG-IMRT) for skull base chordoma and chondrosarcoma: preliminary outcomes. *Neuro Oncol*.

[23] Weber DC, Rutz HP, Pedroni ES, Bolsi A, Timmermann B, Verwey J, Lomax AJ, Goitein G (2005). Results of spot-scanning proton radiation therapy for chordoma and chondrosarcoma of the skull base: the Paul Scherrer Institut experience. *Int J Radiat Oncol Biol Phys*.

[24] Indelicato DJ, Flampouri S, Rotondo RL, Bradley JA, Morris CG, Aldana PR, Sandler E, Mendenhall NP (2014). Incidence and dosimetric parameters of pediatric brainstem toxicity following proton therapy. *Acta Oncol*.

[25] Haas-Kogan D, Indelicato D, Paganetti H, Esiashvili N, Mahajan A, Yock T, Flampouri S, MacDonald S, Fouladi M, Stephen K, Kalapurakal J, Terezakis S, Kooy H, Grosshans D, Makrigiorgos M, Mishra K, Poussaint TY, Cohen K, Fitzgerald T, Gondi V, Liu A, Michalski J, Mirkovic D, Mohan R, Perkins S, Wong K, Vikram B, Buchsbaum J, Kun L (2018). National Cancer Institute Workshop on Proton Therapy for Children: considerations regarding brainstem injury. *Int J Radiat Oncol Biol Phys*.

[26] McDonald MW, Linton OR, Calley CS (2015). Dose-volume relationships associated with temporal lobe radiation necrosis after skull base proton beam therapy. *Int J Radiat Oncol Biol Phys*.

[27] Kitpanit S, Lee A, Pitter KL, Fan D, Chow JCH, Neal B, Han Z, Fox P, Sine K, Mah D, Dunn LA, Sherman EJ, Michel L, Ganly I, Wong RJ, Boyle JO, Cohen MA, Singh B, Brennan CW, Gavrilovic IT, Hatzoglou V, O'Malley B, Zakeri K, Yu Y, Chen L, Gelblum DY, Kang JJ, McBride SM, Tsai CJ, Riaz N, Lee NY (2020). Temporal lobe necrosis in head and neck cancer patients after proton therapy to the skull base. *Int J Part Ther*.

[28] Pehlivan B, Ares C, Lomax AJ, Stadelmann O, Goitein G, Timmermann B, Schneider RA, Hug EB (2012). Temporal lobe toxicity analysis after proton radiation therapy for skull base tumors. *Int J Radiat Oncol Biol Phys*.

[29] Sun Y, Zhou GQ, Qi ZY, Zhang L, Huang SM, Liu LZ, Li L, Lin AH, Ma J (2013). Radiation-induced temporal lobe injury after intensity modulated radiotherapy in nasopharyngeal carcinoma patients: a dose-volume-outcome analysis. *BMC Cancer*.

[30] Zhou GQ, Yu XL, Chen M, Guo R, Lei Y, Sun Y, Mao YP, Liu LZ, Li L, Lin AH, Ma J (2013). Radiation-induced temporal lobe injury for nasopharyngeal carcinoma: a comparison of intensity-modulated radiotherapy and conventional two-dimensional radiotherapy. *PLoS One*.

[31] Su SF, Huang Y, Xiao WW, Huang SM, Han F, Xie CM, Lu TX (2012). Clinical and dosimetric characteristics of temporal lobe injury following intensity modulated radiotherapy of nasopharyngeal carcinoma. *Radiother Oncol*.

[32] Shih AR, Cote GM, Chebib I, Choy E, DeLaney T, Deshpande V, Hornicek FJ, Miao R, Schwab JH, Nielsen GP, Chen YL (2018). Clinicopathologic characteristics of poorly differentiated chordoma. *Mod Pathol*.

